# Electronic health records in Brazil: Prospects and technological challenges

**DOI:** 10.3389/fpubh.2022.963841

**Published:** 2022-11-03

**Authors:** Ingridy M. P. Barbalho, Felipe Fernandes, Daniele M. S. Barros, Jailton C. Paiva, Jorge Henriques, Antônio H. F. Morais, Karilany D. Coutinho, Giliate C. Coelho Neto, Arthur Chioro, Ricardo A. M. Valentim

**Affiliations:** ^1^Laboratory of Technological Innovation in Health (LAIS), Federal University of Rio Grande do Norte (UFRN), Natal, RN, Brazil; ^2^Department of Informatics Engineering, Center for Informatics and Systems of the University of Coimbra, Universidade de Coimbra, Coimbra, Portugal; ^3^Departamento de Medicina Preventiva, Escola Paulista de Medicina, Universidade Federal de São Paulo, São Paulo, SP, Brazil

**Keywords:** electronic health record, electronic medical record, electronic personal health record, healthcare, global health, health record, digital health in Brazil

## Abstract

Electronic Health Records (EHR) are critical tools for advancing digital health worldwide. In Brazil, EHR development must follow specific standards, laws, and guidelines that contribute to implementing beneficial resources for population health monitoring. This paper presents an audit of the main approaches used for EHR development in Brazil, thus highlighting prospects, challenges, and existing gaps in the field. We applied a systematic review protocol to search for articles published from 2011 to 2021 in seven databases (Science Direct, Web of Science, PubMed, Springer, IEEE Xplore, ACM Digital Library, and SciELO). Subsequently, we analyzed 14 articles that met the inclusion and quality criteria and answered our research questions. According to this analysis, 78.58% (11) of the articles state that interoperability between systems is essential for improving patient care. Moreover, many resources are being designed and deployed to achieve this communication between EHRs and other healthcare systems in the Brazilian landscape. Besides interoperability, the articles report other considerable elements: (i) the need for increased security with the deployment of permission resources for viewing patient data, (ii) the absence of accurate data for testing EHRs, and (iii) the relevance of defining a methodology for EHR development. Our review provides an overview of EHR development in Brazil and discusses current gaps, innovative approaches, and technological solutions that could potentially address the related challenges. Lastly, our study also addresses primary elements that could contribute to relevant components of EHR development in the context of Brazil's public health system.

**Systematic review registration:** PROSPERO, identifier CRD42021233219, https://www.crd.york.ac.uk/prospero/display_record.php?ID=CRD42021233219.

## Introduction

Brazil has made sustained efforts to develop and implement information and communications technologies (ICTs) to meet health policy, network, and service needs ever since the first Health Information Systems (HIS) began to emerge in the country (1). Globally, related initiatives have been conventionally referred to as digital health (2) or digital health transformation (3). Current research in this line explores transdisciplinary health knowledge, artificial intelligence, biomedical engineering, bioengineering, and bioinformatics to seek solutions and optimize health processes and services. These include clinical studies, diagnostic and prognostic support, therapeutic and treatment regimens, surgery, and health data management (4).

Developing digital health solutions is a demanding task (5–8). Several countries, meanwhile, have been formulating and implementing their own initiatives as a resource for adding value to health care services (9, 10). Historically, the Brazilian government assumed that role by creating a HIS of national scope (11), and more recently, by implementing technological solutions focused on health data integration and interoperability (12). Renewed emphasis has placed on the development of solutions after the National Health Informatics and Information Policy (PNIIS, its Portuguese acronym) was introduced, an instrument that strongly promotes both aspects (13).

Against this background, electronic health records (EHR) are alternative solutions with the potential to improve care and support public health intervention planning. They permit the collection and exchange of information under specific pre-established semantic and technological standards (14, 15). In addition, EHRs allow the organized collection of patient information and are beneficial for future care provision (16, 17).

EHRs are repositories storing personal health information in an electronically processable form (18, 19) and can either receive data from medical records or from other HIS (e.g., surveillance, laboratory report, medical imaging, and care regulation systems) (20). Such repositories are valuable to healthcare facilities since physicians and other practitioners can benefit from their functionalities (21), which include grouping together the information necessary to ensure patient care and treatment continuity (22–25). In sum, the main goal of EHRs is to provide health care providers, whenever needed, with the patient's clinical data systematized.

Moreover, EHRs are designed to facilitate secure information sharing among the several practitioners operating in different settings (primary health care and medium and high complexity services) (26). EHR information can be readily accessed and updated by authorized personnel, for instance, when a patient undergoes a medical procedure and their information is recorded in the repository (27). Of important note, patient information (e.g., laboratory tests, physical examinations, diagnostic, treatment regimen, etc.) are sensitive and confidential (28). Therefore, allocating resources to protect patient information and prevent data breaches is indispensable. That way, patients will remain reassured about the availability of their health data (29, 30).

Perhaps the main challenge for EHR implementation is integrating legacy systems that, albeit created in a fragmented and unstandardized fashion, store data of services delivered to each patient in health centers throughout their lifetime. The lack of interoperability standards implementation in EHRs adopted makes it complex to centralize or know the patient's medical history data, thus hindering its continuous construction (31). And this issue is compounded by situations in which patients needing multidisciplinary monitoring have their information collated by several practitioners. Hence, monitoring disease courses becomes even more detrimental (32, 33).

Data fragmentation due to legacy systems (non-interoperable) also hampers epidemiological assessment, which is critically important under outbreak, epidemic, and pandemic scenarios (13). Albeit standards to maintain optimal interoperability with EHRs have been defined, achieving a satisfactory level of integration remains a demanding task (34, 35). Pfeuffer et al. (36) claim that interoperability, security, privacy, low acceptance by physicians, and issues alike constitute the main obstacles to implementing an EHR.

From that standpoint, mobilizing technological resources to overcome such barriers is essential to achieving a plausible scenario in which patients' health information is securely stored and shared (37, 38). The implementation of digital health strategies can strengthen the health system and provide desired benefits in terms of care, management, and organization at the existing levels (39–41). In that respect, Brazil has made significant advances during the COVID-19 pandemic after implementing the National Health Data Network (RNDS, its Portuguese acronym), thereby creating a more favorable environment for interoperability (42). Yet several challenges loom as several outdated and fragmented systems continue to be part of the Brazilian National Health System (SUS, its Portuguese acronym) structure (11).

In this context, we conducted a Systematic Literature Review (SLR) on the main approaches applied to EHR development in Brazil. Our paper presents (i) the technological resources, architectures, and standards used for EHRs development, (ii) the organization and structuring of EHRs for data collection; (iii) a brief discussion of the contributions and evolution of EHRs development in Brazil. In addition, we highlight current perspectives, challenges, and gaps in this field.

### Digital health in Brazil

Although Brazil has a history of bureaucratic development and deployment of health information systems (43), this country's health facilities have undergone a digital transformation (44, 45). Data available from the survey on ICT in Brazilian facilities, conducted annually by the Brazilian Network Information Center (Nic.Br), provides results that allow to map out digital health in Brazil and prepare the health system for ICT incorporation in the sector (43).

Indicators from 2019 show that 82% of health facilities have adopted electronic systems for recording information. In 2018, this number was 73%. The country has available the resources required to optimize technological platforms' production and therefore raise those figures. The PNIIS is an initiative of the Brazilian Ministry of Health (MoH) to promote ICT adoption and, as a result, enhance work processes in health. Its key focus is to offer a National Health Information System (SNIS, for its acronym in Portuguese) capable of providing citizens with information and managing and producing knowledge and social control. Moreover, a system that foments equitable, comprehensive, and humanized health services and promotes health system effectiveness and quality by expanding access to health (19). HIS are powerful tools that allow for timely decision-making by the several management levels; however, data must be first qualified by robust models of integrity.

The Federal Government's support has greatly underpinned this digital transformation by making available large-scale HIS used by state and local health departments. The Health Information System for Primary Care (SISAB, its Portuguese acronym) (46), established through a 2013 ministerial decree, electronically records data on consultations and activities. Presently, SISAB constitutes the predominant HIS in Primary Health Care (PHC) in Brazil. Available in virtually every Brazilian municipality, it has been used for funding and adherence purposes concerning the programs and strategies laid out in the National Primary Care Policy (PNAB, its Portuguese acronym).

The Brazilian National Regulatory System (SISREG, its Portuguese acronym) (47), which manages the flow of SUS users in health care networks (e.g., scheduling specialty care appointments), is used in more than 2,000 municipalities on a daily basis. The National Pharmaceutical Assistance System (known by its Portuguese acronym, Hórus system), responsible for the logistics of controlling pharmaceutical stocks and drug dispensing, is used in more than 1.5 public pharmacies. Such systems, however, are generally not integrated among them or with states, municipalities, and the private sector (48).

To overcome this issue, the MoH has established technological and semantic standards. Hence, Ministerial Order No. 2,073 of 2011 (49) regulates the use of interoperability standards and health information for those systems, indicating the main standards that can or should be adopted. Table 1 lists such standards and their respective descriptions (50).

**Table 1:** Interoperability standards set forth in the Ministerial Order No. 2,073 of 2011.

**Standard**	**Description**
OpenEHR	Reference model for EHR definition
HL7	Standard for maintaining system interoperability
HL7 CDA	Clinical document architecture
SNOMED-CT	Nomenclature of clinical terms
LOINC	Nomenclature and coding of laboratory tests
TISS	Interoperability among supplementary health systems
DICOM	Standard for imaging exams-related information
ISO 13606-2	Knowledge models under archetype and template form, and management methodologies

HL7, Health Level 7; CDA, Clinical Document Architecture; SNOMED-CT, Systematized Nomenclature of Medicine-Clinical Terms; LOINC, Logical Observation Identifiers Names and Codes; TISS, Exchange of Information in Supplementary Health; DICOM, Digital Imaging and Communications in Medicine; ISO, International Organization for Standardization.

Aiming to improve HIS standardization, especially with respect to EHRs, the Federal Council of Medicine (CFM, its Portuguese acronym) and the Brazilian Society of Health Informatics (SBIS, its Portuguese acronym) established a technical and scientific cooperation agreement in force since 2002. Such cooperation set forth norms, standards, and regulations for EHR development (51) and has led to the creation of an EHR Systems Certification process. By defining compulsory requirements under the federal legislation for electronic documents, such a process reinforced the mandatory use of digital certification (electronic signature) for EHRs ethical and legal validity (52).

Other Brazilian organizations are also interested in and contribute to developing and implementing EHRs. These include the Brazilian Health System Informatics Department (DATASUS, its Portuguese acronym) of the Brazil's MoH, the National Council of Health Secretaries (CONASS, its Portuguese acronym), the National Council of Municipal Health Secretariats (CONASEMS, its Portuguese acronym), the National Health Council (CNS, its Portuguese acronym), the Department of Digital Health (DESD, its Portuguese acronym), the Brazilian Telehealth Center, among others. In recent years, such organizations have been engaged in discussions about the impact and importance of adopting EHRs for advancing digital public health in the country.

In 2019, Brazil implemented the RDNS, a health data interoperability platform designed to promote information exchange between services across health care networks. Besides creating an integrated ecosystem for SUS health information systems, the RNDS enables the transition and continuity of care between public and private sectors (12). This strategy includes the concept of a modern platform with norms providing innovative interoperability and connectivity between systems. In addition, since 2011, the MoH has been exploring alternative ways (53) to improve the integration of data operated by end-users, i.e., the citizen/patient, through “single window” type solutions (54). And “Connect SUS” is a case in point.

Connect SUS is a national health platform for citizens, health practitioners, and managers (55). Its purpose is to integrate the citizen's health information into an extensive data network, thus providing health practitioners and managers with access to a wide range of health data with the potential to improve the continuity of care and decision-making (42, 56). It is a platform for citizens to access their health information based on their health care records in the SUS and private services, including tests, appointments, vaccinations, and medication discontinuation. Connect SUS implementation is expected to improve health services delivered to the population.

Given this scenario, it becomes noticeable that many digital health solutions are already in force, and many others are being strategically planned in search of improving health services. To validate the implemented modifications, such solutions must be monitored, evaluated, and restructured to meet the workflow needs better, thus making this process long and continuous. Ultimately, investments in work processes, technologies, people, policy-making, and equipment are vital to maximizing the positive impacts of digital health (57–59).

## Methods

This SLR is based on the systematic review guidelines proposed by Kitchenham (60) and follows the Preferred Reporting Items for Systematic Reviews and Meta-Analyses (PRISMA) checklist (61). Moreover, we registered this review in the International prospective register of systematic reviews (PROSPERO) (62), under registration No. CRD42021233219 (63).

This study sought to investigate EHR development in Brazil and identify major approaches and gaps in this context. Given this premise, we designed Research Questions (RQs) (see Table 2) to compile relevant information.

**Table 2:** Research questions.

**RQs**	**Description**
01	What is the approach being discussed?
02	What is the research problem mentioned?
03	Does the presented EHR follow the Brazilian Ministry of Health protocols?
04	What technology/resource was used to solve the problem under discussion?
05	Does the paper address any standards Brazil has adopted for EHR development?

We searched and selected papers published between 2011 and 2021 in Science Direct, Web of Science, PubMed, Springer, IEEE Xplore, ACM Digital Library, and SciELO databases using the search terms (“electronic medical record” OR “electronic patient record” OR “electronic health record”) AND (Brazil OR Brasil). Thus, it consisted of three steps: (i) paper identification and organization, (ii) paper triage through quality screening (inclusion and exclusion criteria), and (iii) paper analysis according to quality criteria. In step (i), we selected the first set of articles the database search returned.

In step (ii), we defined and applied three inclusion criteria (IC) and four exclusion criteria (EC) (see Table 3) to the initial set of papers selected in step (i). Then, those articles directly related to this systematic review's focus area were chosen for analysis. Before the protocol for this review was set, we analyzed the conference papers. Study maturity level and methodological and technological approach considered, we opted only to explore articles published in journals. After paper screening based on the IC, we applied the EC, thus checking for and removing duplicate papers. We conducted a further filtering process by screening the title, abstract, and keywords to remove ineligible papers, i.e., any papers without specific terms of interest for this review. In addition, studies not relating to EHR development in Brazil were not considered. This screening was performed using the Rayyan (64) web application. After this step, all articles were reviewed by three authors.

**Table 3:** Inclusion and exclusion criteria.

**N**	**IC**	**EC**
01	Articles published between 2011 and 2021	Duplicate articles
02	Research articles published in journals	Secondary articles, review articles, or articles published in conference proceedings
03	Articles in the fields of technology, engineering and/or computer science	Articles covering epidemiological studies or using electronic health record data
04	–	Studies unrelated to EHR development in Brazil

In step (iii), the eligible articles were fully read according to the Quality Assessment (QA) protocol and its criteria (see Table 4). During QA, a score measuring the paper's relevance to this review was assigned for each criterion. This score is distributed in weights for possible answers to the QA criteria included in the primary studies, with 1.0 being the most relevant weight and 0 being the least.


$$QA=\{\begin{array}{c} \text{1}.\text{0 },\text{yes},\text{ it fully describes},\\ \text{0}.\text{5},\text{yes},\text{ it partially describes},\\ \text{0},\text{ it does not describe}. \end{array}$$

**Table 4:** Quality criteria.

**QA**	**Description**
01	Does the paper clearly state the research purpose?
02	Does the study address issues involving EHRs development in Brazil?
03	Does the paper examine technologies used in the development of EHRs?
04	Does the paper address the contribution to the development of EHRs?

For each paper, a score (Equation 1) was calculated using the arithmetic mean of the QA criteria scores (Table 4). Thus, all papers scoring greater than or equal to 0.5 (0.5 ≤ score) were selected for this research and now comprise the last set of papers.


(1) $$\begin{array}{l} score\;=\;\frac{1}{n}\overset{n}{\underset{i=1}{\sum }}QA_{i} \end{array}$$

The records relative to each step, along with the data extracted from the papers, were adequately registered in a spreadsheet and stored on Rayyan. Details such as publication year, authors, and potential answers to the RQs were extracted from the set of papers selected in step (iii). Such answers allowed us to perform the final analysis and achieve this systematic review's purpose.

## Results

**Figure 1:**
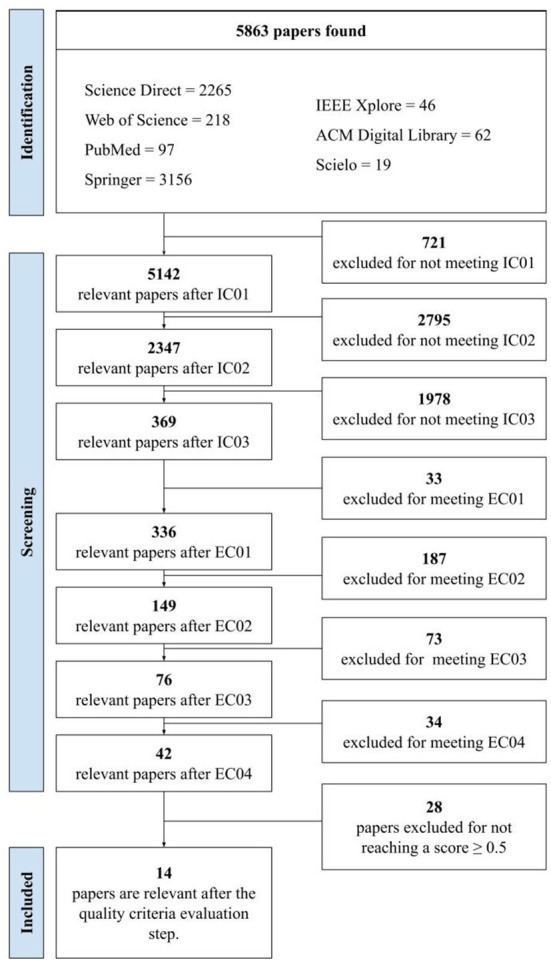
Flowchart adapted from the PRISMA with result of the execution of the systematic review protocol.

The results obtained through the search protocol are shown in Figure 1. As of November 2021, when step (i) was performed, 5,863 papers were retrieved from the consulted databases according to the search terms selected. Subsequently, this set of papers underwent quality screening. In this step, there was a refinement after applying the CI-based items (Table 3), which excluded 5,494 articles for not meeting such criteria. After this refinement, 369 articles were considered suitable for the next step. In this analysis, the CE-based filters (Table 3) excluded 327 articles, totaling 42 articles for the entire reading and evaluation based on the AQs. The detailed analysis and assignment of the AQ values were discarded in 28 papers from the search for not reaching the defined score (≥0.5).

Regarding the articles excluded, 60.71% (17) did not address technical concepts about the development of EHR, 25% (7) had content related only to the use of EHR, and 10.71% (3) were not associated with research on EHR in Brazil, and 3.58% (1) were not available for reading. Finally, 14 articles were assessed for this systematic review's final analysis and investigation.

**Figure 2:**
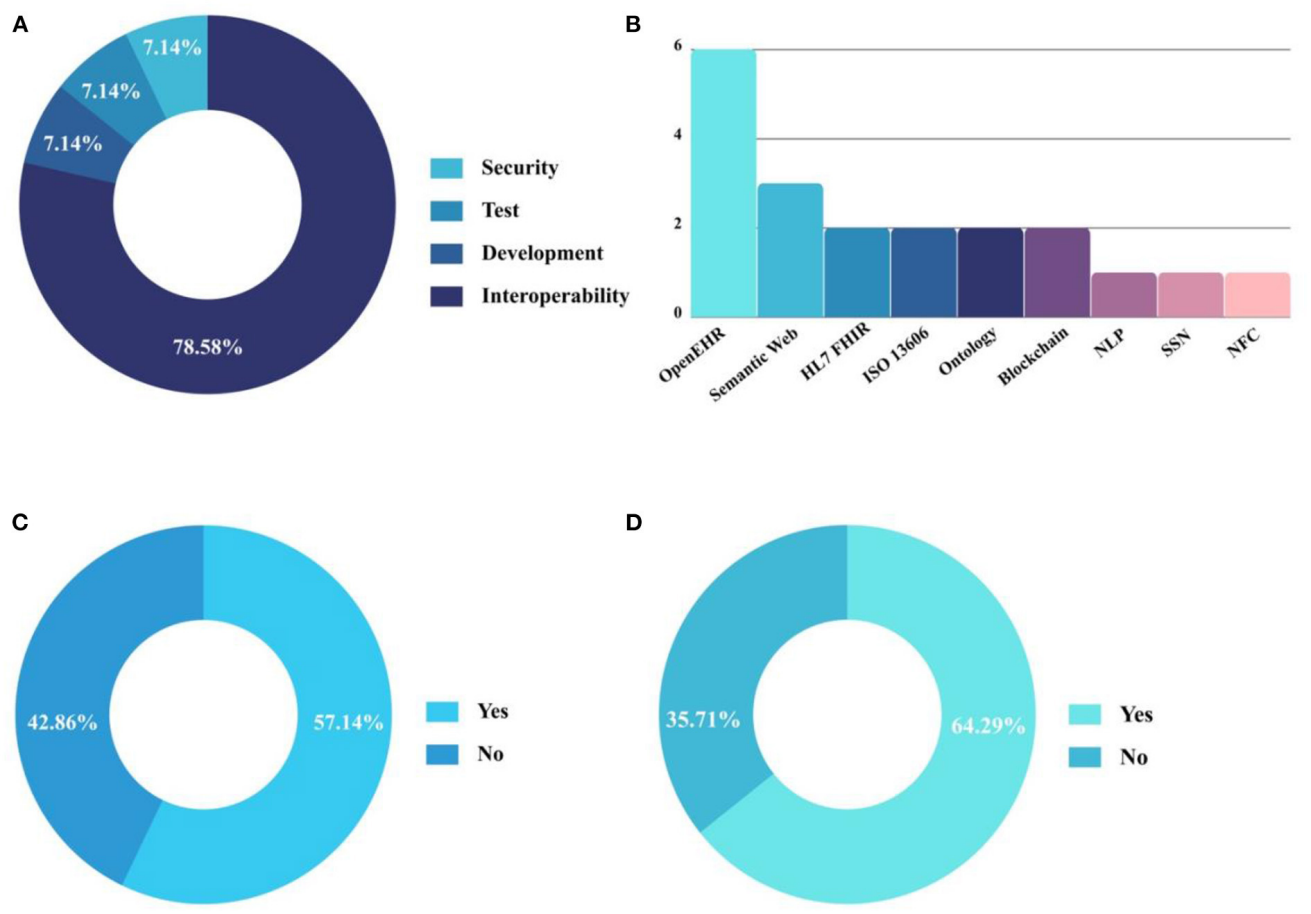
Analysis of accepted articles after performing the systematic review protocol. **(A)** Approach discussed; **(B)** Standards and technologies used; **(C)** Percentage of EHR developed according to the Brazilian Ministry of Health; **(D)** Percentage of EHR developed using standards established by Brazilian guidelines.

Thus, engaging in a more comprehensive analysis of the 14 selected articles, it was possible to highlight four different approaches related to the development of EHR in Brazil, as shown in Figure 2A. Most articles report the need to implement interoperability between EHR and other HIS. Besides interoperability, the other publications report the need for permission functionalities to visualize patient data, the absence of real data to perform data persistence tests in EHR, and the importance of defining a methodology for the development of EHR.

Of the studies analyzed, 57.14% (8) present the development of the EHR based on the documentation used by the Ministry of Health for conducting consultations and/or patient follow-up (Figure 2C) and 64.29% (9) of the EHRs analyzed were developed using some of the standards established by the Brazilian guidelines addressed in Ordinance N° 2.073 (Figure 2D). Figure 2B presents the leading technologies and standards used for the development of EHR. Other significant features extracted from the 14 articles included in this study are summarized in Table 5 to support the analysis and answer the research questions. The included articles were published during the following years: 2012 (*n* = 1), 2015 (*n* = 1), 2017 (*n* = 2), 2018 (*n* = 2), 2019 (*n* = 6), and 2020 (*n* = 2).

**Table 5:** Characteristics of the selected set of article.

**Reference**	**Score**	**Approach**	**Problem**	**Goal**	**QP04**	**QP05**
Santos et at. (65)	1	Interoperability	Diversity of data models and terminologies and concepts adopted in EHR development	Introduce the archetype modeling process to support the building of your regional EHR system	OpenEHR, ISO 13606	Yes
Maia et al. (66)	0.5	Interoperability	Lack of development methodology for the archetypes used in the EHR medical record in SUS	Define the steps, functions, and artifacts of the archetype governance process used in local EHR	ISO 13606	Yes
Pahl et al. (67)	0.875	Interoperability	The currently existing archetypes do not cover clinical and demographic data related to obstetrics	Investigating whether openEHR is a possible approach for modeling HIS at the regional level	OpenEHR	Yes
Souza et al. (68)	1	Interoperability	Absence of technical interoperability solutions between EHR systems for public health associations in Brazil	Propose an architecture to provide technical interoperability between EHR systems in Brazilian public health organizations	SOA, HL7	Yes
Pellison et al. (69)	0.875	Interoperability	Need for data integration and integrity for optimal and desirable health care	Describe the research methods used to develop a system that uses interoperability techniques based on the Semantic Web	Semantic Web	No
Lima et al. (70)	0.75	Interoperability	Lack of features to protect unauthorized users from accessing patient data	Implement security mechanisms for an API that allows data extraction from a regional healthcare system	Semantic Web	No
Crepaldi et al. (71)	0.625	Interoperability	The diversity of non-interoperable systems to aid in the treatment of a disease	Development of an interoperable ecosystem and a decision support model to aid in the treatment of tuberculosis	Semantic Web and Ontology	No
Roehrs et al. (72)	1	Interoperability	Having a unified view of patients' health history	Provide a distributed, interoperable architecture model for PHR that addresses a unified point of view for patients and healthcare professionals	OpenEHR	Yes
Roehrs et al. (73)	1	Interoperability	Get a unified view of health data distributed among different healthcare providers	Implement and evaluate a PHR model that integrates distributed health records	Blockchain	Yes
Roehrs et al. (74)	1	Interoperability	Difficulty of integrating PHR delimiting a unified view of patient data in the face of multiple existing patient health data standards	Evaluate the semantic interoperability structuring and integration of different healthcare standards	OpenEHR, HL7 FHIR, ontology, NLP	Yes
Rubí and Gondim (75)	1	Interoperability	Lack of interoperability standards between IoMT platform and RES	Definition of an interoperable IoMT platform through the joint use of openEHR semantics and SSN	Ontology (openEHR and SSN)	Yes
Quincozes and Kazienko (76)	0.75	Secure data access	Provide secure access to patient information on ubiquitous EHR systems	Use ubiquitous computing to improve EHR access management	NFC	No
Teodoro et al. (77)	1	Test with real data	Test with real data & Absence of publicly available health data to test, compare and validate different data persistence mechanisms in the openEHR formalism	Present a large set of real data from DATASUS for more robust testing in EHR based on the openEHR model	OpenEHR	Yes
France et al. (78)	0.875	Software Development	Legacy integration and the absence of a system architecture view	Develop an EHR system using SOA principles and techniques to gather important information from legacy systems	SOAQM	No

QP04, What technology/resource was used to solve the problem under discussion?; QP05, Does the paper address any standards Brazil has adopted for EHR development?

### Interoperability

Currently, there is a strong trend toward using clinical information models to promote interoperability between EHR. This modeling consists of defining specific concepts and archetypes promoted by healthcare professionals. The process of developing archetypes is a type of knowledge management that aims to facilitate the understanding and development of EHR systems that meet the needs of health professionals and the continued care of the citizen. Santos et al. (65) assert that archetypes are important semantic artifacts for achieving semantic interoperability between EHR systems. To prove such an assertion, the authors present the steps of the archetype modeling process to support the construction of an interoperable regional EHR system. The decision to use archetypes was based on the flexibility and easiness these artifacts can offer when reusing medical knowledge. The authors judge that the ISO 13606 reference model, together with the definition of archetypes, is sufficient to meet the specificities for creating a regional EHR system for primary health care in a federative unit of Brazil. These are only examples of possible headings. Please feel free to use different headings to best describe your results.

A few years later, Maia et al. (66) presented a similar study, proposing a model for the process of creating archetypes that can subsidize the development of EHR, considering the legislation of the Unified Health System in Brazil. The model has steps, functions, and primary artifacts for the governance process of the archetype to be used in the interoperable EHR of health care networks. In addition, quality requirements were defined to address the clinical, public health management, technical, and governance information of the archetype development process used when creating the model. This process can significantly increase the coherence between EHR systems and the public policies established in Brazil, supporting the organization of public health information in a scenario with the initial implementation of EHR.

Pahl et al. (67) investigated the feasibility of using openEHR for the digital representation of demographic and clinical data required by the Ministry of Health regarding obstetric follow-up in Brazilian health units. The investigation, modification, and implementation of new archetypes, according to the openEHR formalism, were performed on the Clinical Knowledge Manager (CKM) platform using the LinkEHR archetype editor. The study highlights that CKM provides archetypes to represent a particular data snippet and can represent missing information in a given context. The results further reinforce confidence that the openEHR approach is highly usable for international representation of clinical and demographic data and also confirm that openEHR is sufficient for national-level data representation in the context of the digital transformation of primary health care in Brazil.

Souza et al. (68) stated that even with the definition of standards, there is still a deficiency when it comes to technical interoperability solutions between EHR systems for public health organizations in Brazil. The authors present an architecture capable of providing technical interoperability between EHR systems in public health organizations based on an evaluation of different interoperability architectures proposed in the literature. The proposed architecture was based on the Social and Health Information System of the Lombardy Region, in Italy, and some adaptations were made to meet Brazilian requirements and legislation. The adoption of an SOA-based architecture promoted interoperability among electronic record systems. In addition, this architecture integrates different applications using the HL7 message exchange standard. The main purpose of this suggestion was to ensure that administrative data at the healthcare organization level is synchronized with a central citizen registry, which contains up-to-date information on all citizens in the region, to avoid erroneous or duplicate data. A use case scenario was also presented, where it was possible to see the feasibility of applying architecture in organizations using different EHR.

The lack of standardization in patient data collection considerably hinders the continuation of adequate care, especially for patients who pass through different levels of care. To improve the quality of this process in the context of tuberculosis, Pellison et al. (69) proposed an architecture that uses semantic web standards recommended by W3C (World Wide Web Consortium) as interoperability tools for reducing the costs of records storage and management. The proposed architecture expects to provide a single, organized, and complete database of the assisted population. The authors intend to evaluate the impact of integration and interoperability methods between systems from e-SUS Primary Care (e-SUS AB, in Portuguese: e-SUS Atenção Básica), System for Tuberculosis (SisTB), Notification and Monitoring System for Cases of Tuberculosis in the State of São Paulo (TBWEB, its Portuguese acronym) and information systems from other hospitals. The evaluation metrics related to data quality include completeness, consistency, duplicity, accuracy, and management metrics.

The semantic web was also a topic of research in the work conducted by Lima et al. (70). They presented the implementation of security mechanisms for a semantic API that allows the extraction of health data related to tuberculosis cases from a regional health information system called SisTB, designed to generate notifications and track patients diagnosed with tuberculosis. The proposal establishes access levels for exchanging health data between systems using the semantic API. Through the established access levels, the authors believe it will be possible to semantically segment the tagged content in SisTB to allow access only to specific systems with an appropriate access level. Systems that do not meet the access level defined by the API will not be allowed to view the information at that level. Systems that do not integrate the ecosystem will be able to access the data through identification and authentication through public/private keys. Thus, the proposal presents an alternative for data sharing, considering aspects related to semantic interoperability, security, and access authorization to the data stored in the system.

Crepaldi et al. (71) presented the project of the SisTB ecosystem, which is formed by a set of systems and applications designed to assist in the treatment of tuberculosis and improve the routine of health professionals regarding the monitoring of their patients. The ecosystem was designed based on the need to store and consult information and make it available quickly and easily for reporting, as recommended by the Brazilian Ministry of Health. The SisTB ecosystem has layers to manage and interpret the information available from the other systems. The interoperability and security layers are responsible for the ability to exchange data between SisTB and other systems securely. The interoperability layer has its base on standards recommended by the W3C and the semantic web paradigm. The semantic interoperability can be achieved by defining and using a domain-specific ontology. This ontology represents the concepts and specificities of tuberculosis in the Brazilian context. On the other hand, reaching functional interoperability through communication protocols, APIs, and semantic query endpoints, such as SPARQL endpoints. The security layer provides robust methods for transferring data between authorized applications over the Internet. With this, the development of the SisTB ecosystem intends to minimize the problem related to the use of several isolated information systems without interoperability, which can impair patient follow-up.

From the perspective of improving access to patient data stored in multiple locations, Roehrs et al. (72) proposed a distributed and interoperable architecture model, called OmniPHR, which uses blockchain technology and the openEHR interoperability standard to integrate patient health records. One of the main goals of this architecture is to provide interoperability between different healthcare providers by enabling access to patient health records. OmniPHR proposes using a P2P network to represent a hierarchically organized, encrypted, and distributed Personal Health Record (PHR) in data blocks chained over the network. The proposed architecture contributes to secure sharing since the authors present strategies to promote the unification of patient health data.

The evaluation of the information exchange security of the architecture proposed by Roehrs et al. (72) was performed from the implementation of OmniPHR Roehrs et al. (73). The authors used real data to test the blockchain network implementation to achieve this goal, measuring the architecture's performance in several concurrent access scenarios. The evaluation sought to reflect the splitting, replication, and communication of data blocks in the network. The results indicated that combining the openEHR standard with blockchain technologies created a unified and interoperable view of healthcare data. Thus, the implementation of the OmniPHR model showed that it is possible to integrate distributed data into a unified view of patient health records, making up-to-date patient health information available to improve the quality of care.

Still using the same proposal, Roehrs et al. (74) evaluate the interoperability and integration structure of the OmniPHR prototype using different healthcare standards currently adopted. The analyzed model has the organizational domain, which aims to maintain the original data contained in the healthcare providers' databases, and the personal domain consists of a middleware with the inclusion of repositories where the PHR is stored. The organizational domain provides for the input of open and legacy standards. To represent the open standards, openEHR and HL7 FHIR were used. The reference model Medical Information Mart for Intensive Care (MIMIC-III) was used to evaluate the legacy standards. The middleware is composed of a translator component in the personal domain, making it possible to receive data in any of the three formats mentioned above. Upon receiving the data, the translator component reads and converts this data into the openEHR ontology through a Natural Language Processing (NLP) algorithm. The authors state that it is possible to integrate different patterns by using the ontology, allowing inferences to be made from this data. The research results demonstrated the possibility of a unified and updated view of PHR data for patients and healthcare professionals, presenting a solution based on artificial intelligence with NLP, ontology, and an open healthcare standard to achieve semantic interoperability. Moreover, this proposal contributes to obtaining original data from different standards in a single format.

In addition to the use of EHR, the Internet of Medical Things (IoMT) platform is revolutionizing patient care and monitoring. The continuous and detailed information enables healthcare professionals to provide more accurate services, improving patients' quality of life. However, it is necessary for the data produced by IoMT devices to be sent to the respective patient's EHR. However, integrating data from IoMT platforms with EHR is not a simple task and presents some challenges, such as the communication standards and data models that are produced. Trying to solve this problem, Rubí and Gondim (75) proposed the joint use of openEHR semantics with Semantic Sensor Network (SSN) to achieve interoperability at the semantic level and the use of a machine-to-machine (M2M) architecture for the definition of an interoperable IoMT platform. The main contribution of the platform is the development of an ontology that aligns the healthcare domain (openEHR) with the IoMT technical domain (SSN). This ontology serves as a model for data storage following a semantic web approach capable of identifying sensors and automatically translating the detected data into Web Ontology Language (OWL) individuals, thus ensuring semantic coherence between the two domains. Another relevant aspect, concerns the semantic extension of the openEHR model to the M2M domain, which enabled definitions of heterogeneous IoMT devices within a single data model. The study favors the development of modern healthcare services, with interoperability between different devices that compose an IoMT platform and the EHR.

### Data visualization security

Providing secure access to patient information in ubiquitous EHR systems is not a simple task. It is essential that the OR has features to prevent intruders, through impersonation, from gaining access to the devices used to access and update patient data. One of the biggest challenges is to ensure device authentication by avoiding impersonation. To address this problem, Quincozes and Kazienko (76) analyze a secure architecture based on ubiquitous computing, proposed by Quincozes and Kazienko (79), for retrieving and maintaining medical records. The authors show the feasibility of using devices (smartphones, laptops, and tags) for patient monitoring while maintaining secure access to EHR information on a local network. The analyzed architecture uses Near Field Communication (NFC) technology for patient identification. The healthcare professional uses a device to scan the NFC tag under the patient's possession to access patient information. The experiments performed show that the system implemented had high usability among healthcare professionals and demonstrated that the architecture is a viable alternative to prevent intruders from accessing the devices and, consequently, the patient's confidential information.

### Testing with real data

The testing stage is fundamental in any software development process, especially for systems intended to store health data. Due to the sensitive content, accessing this data for research purposes, while necessary, is often a complex procedure. Even with a large number of studies, it is currently still difficult to find publicly available health datasets in openEHR format that can be used to test, compare and validate different data persistence mechanisms. To minimize this problem, Teodoro et al. (77) presents a large dataset, called openEHR Benchmark Dataset (ORBDA), coded according to openEHR formalism to promote research and fostering development, offer subsidies for more robust and reliable testing of EHR systems based on the openEHR model. This foundation has the potential to contribute to the engineering, quality improvement, and consequently widespread adoption of openEHR-based electronic health record systems.

### Software development

The insertion of EHR for patient follow-up has been used for a few years now. These systems, considered legacy, have a lot of important information and somehow must keep up with the evolution of technology. França et al. (78) describes the development of an EHR system focused on data integration against a series of legacy systems that store important information about patients, exams, appointments and other data from a public hospital in Brazil. These systems are not integrated, and the need for data replication in different systems is recurrent. Due to the restrictions found in this scenario, the proposed solution consists of using Service-Oriented Architecture (SOA) principles and techniques to gather all the important information from the legacy systems and map them into services to be consumed by the EHR system. In this perspective, the authors propose using a quality model, inspired by ISO/IEC 25010 called Service-Oriented Architecture Quality Model (SOAQM), with multiple views of architecture and software design to guide the EHR system's development process. The proposed model defines the actual applicability of ISO/IEC 25010 quality characteristics and the essential attributes for applications in the SOA context. According to the analysis performed, the EHR development process is guided by the quality of the SOAQM values attributes in software development from the early stages. The model approached emphasizes the importance of the comprehensive definition of the architecture, enabling the definition of essential artifacts to understand and maintain the developed system.

## Discussion

This SLR investigated articles that address technological resources and standards used for EHR development in Brazil. Fourteen articles were included and analyzed after performing the SLR protocol. It was possible to observe the main approaches related to the theme, the technologies and standards used to overcome the existing problems, and the list of documents, norms, and guidelines defined by the Brazilian Ministry of Health to develop EHR.

As the main approach discussed, interoperability is present in 78.58% (11) of the analyzed articles. The exchange of information between HIS is a desirable scenario not only in Brazil, but all over the world (80, 81). Even with standards that securely enable this exchange of information, implementing interoperability is still a challenging task. Semantic interoperability, for example, requires mechanisms that enable the exchange of information and the understanding of information between systems. For this, Santos et al. (65), Pahl et al. (67), Roehrs et al. (72), Roehrs et al. (74), and Rubí and Gondim (75) used the formalism of the openEHR standard to represent, in digital form, the information needed for the development of the EHR. The openEHR consists of open specifications, clinical knowledge models, and software that can be used to create standards and build healthcare information and interoperability solutions (82). Some authors have used openEHR in conjunction with other technologies/resources to promote interoperability (Table 5). On the other hand, openEHR has also been used to formalize a large health database (77). This formalization allows, to the dataset, a generalization in which several systems bound to this modeling can use this base for testing, reducing problems related to the absence of accurate data for testing. Given the results presented, the technology meets those needs related to implementing interoperability in EHR.

In Brazil, to achieve a high level of interoperability, the RNDS was produced. It is a mechanism that allows health information systems to exchange information. None of the articles analyzed uses or addresses the RNDS, whose creation is recent. With the perspective of optimizing digital health in the country, the adherence to the RNDS mechanisms in all EHR present in health facilities in Brazil is expected. Figure 2C shows that 64.29% of the articles analyzed used some of the standards adopted by Brazil for the development of EHR. HL7, even being the standard adopted by Brazil to promote interoperability between the EHR, was used only by Roehrs et al. (74) and Souza et al. (68).

Another relevant aspect in view of the analyses performed consists in the adoption of a minimum data set with the main information that must be collected from each patient. Pellison et al. (69) show in their work that the ecosystem developed is composed of forms made available by the Ministry of Health for the detailed follow-up of tuberculosis patients. The adoption of a minimum data set assists in the standardization of data and can minimize the difficulty of collecting the essential information for the follow-up and evolution of the disease, an aspect that avoids duplicity, inconsistencies, and loss of patient information. This information enables a more comprehensive scenario for the decision-making process.

The adoption of technological resources at the various levels of health care, along with the training of health professionals to use these technologies, can promote an improvement in services, as well as promote a rational and qualified application of resources and management of inputs. These technologies are capable of promoting the sharing of information in a safe way, as a way to unify the patient's medical record and the automatic collection of data. Nowadays, healthcare services rely on the implementation of modern tools that provide several benefits for both healthcare professionals and patients. Smart devices collect data through body sensors that allow remote and continuous monitoring of the patient with more convenience (83–85). When connected to IoMT platforms, these devices generate a large volume of data and a rich set of details about patient health. However, as discussed in Rubí and Gondim (75), the lack of semantic interoperability between EHR and IoMT platforms negatively impacts the development of these services since measurements collected by IoMT platforms can comprise a large volume of heterogeneous data. This information is essential for proper patient follow-up and disease monitoring, especially for rare and poorly understood diseases (86). This aspect becomes increasingly necessary, especially by the impositions in the face of digital health transformation in Brazil and global health.

In cases of complex diseases, the patient must have a multidisciplinary follow-up, which requires secure patient data with the team's professionals. Due to patient data sensitivity, sharing this information must be done through protection mechanisms that ensure access only to authorized users. Security aspects such as authentication, authorization, and encryption must be considered in data sharing. In addition to addressing interoperability, Lima et al. (70), Roehrs et al. (72), and Roehrs et al. (73) presented relevant concepts used to promote security in the face of sharing patient information. Blockchain technology, used in Roehrs et al. (72) and Roehrs et al. (73), are being widely used in healthcare to promote decentralization and secure data sharing (87–92).

Secure visualization of patient data, availability of real data for testing, storage of health data across different organizations, and standardization of data in health records are important factors to look at for the development of EHRs. In the current scenario, the main objective is to make patient health data securely available to promote quality in ongoing clinical care. The lack of implementation of these factors can create challenges for the advancement of digital health, such as the unfeasibility of care provided by a variety of professionals in different health care institutions; the complexity of a unified and comprehensive view of the patient due to data fragmentation among different health care providers; and, the unavailability of data for continuity of care in case of change of health care service. In this context, the analyzed characteristics contribute to the need for an interoperability architecture, presenting relevant aspects that can achieve objectives related to the development of EHR.

For the most part, the works studied and analyzed in this systematic review pointed to the interoperability problem in terms of HIS in Brazil—notably, an aspect that directly impacts the fragmentation and quality of health information. This pressing problem poses many challenges to Brazil because the quality, traceability, monitoring, and evaluation of patient data and information plays a pivotal role in guiding public health policies. Moreover, this phenomenon is directly related to the integration not only of HIS but mainly of two crucial areas for the SUS, which are Health Surveillance and Health Care.

In this context, there are numerous obstacles to interoperability. In Brazil, there is currently a large number of health information systems, many of them obsolete. For instance, the Notifiable Diseases Information System (SINAN) is not available in the RNDS, nor has it been integrated with the systems developed by the Brazilian MoH and many others (11, 48). This scenario is even more critical because the MoH, which is, in the SUS, the reference for the proposals of HIS, often does not recognize the conditions of the technological infrastructure in a country where there is enormous cultural, territorial, social, and economic diversity. The country's political instability and the lack of stable job positions for information technology professionals is also a big issue and therefore constitutes a strenuous challenge.

Such challenges can be overcome over the medium and long term, but for this, it is necessary to guarantee the full participation of states and municipalities. It is fundamentally necessary to include them not only in the planning for the use of new technologies after they are ready but in a top-down model. It is essential to develop a logic of incorporating technologies from the territories. That is, states and municipalities must have the autonomy to create, incorporate their technologies, and then integrate their health information systems with the federal government in a down-top model. An important step has already been taken in this direction through the RNDS. However, it is still necessary to define the minimum data models for the diverse demands of the SUS, this is not a trivial task in a country as complex as Brazil, but it is necessary.

Finally, it is essential to highlight poor communication between information technology professionals, policy-makers, and health professionals in Brazil. Articulated and synergistic communication within this triad is necessary for including new interoperable technologies that can meet the needs of the SUS more effectively, especially for managing more qualified information. It is noteworthy that the poor communication among these actors is a recurrent problem in the country, often compromising some initiatives, however brilliant they may be from a computational perspective.

The problems faced when producing accurate, complete, and real-time information during the COVID-19 pandemic are a good example of these significant obstacles. However, these issues could be overcome by developing, incorporating and integrating technologies into the SUS and with well-articulated technical cooperation among the various actors. The Technological Ecosystem for COVID-19 Response in the SUS is a case in point. It was developed and implemented in the State of Rio Grande do Norte in Brazil, during the COVID-19 pandemic (13). In this instance, in addition to technical cooperation actions and effective communication, the parties involved also prioritized transparency and social control, which served as inductive mechanisms for adhering (mitigated resistance) to online technologies capable of providing more timely data and information.

### Conclusions

The study addressed in this paper presented a comprehensive overview of the use and development of EHR in Brazil, highlighted and discussed the problems related to the theme, and possible solutions. The results point to interoperability as a necessary aspect. In fact, this is a fundamental point, especially in the global health context, which will require a greater interaction HIS by sharing data safely and reliably. Other important factors for the development of EHRs are related to the application of technology to improve the security of data visualization and sharing, the use of real data for testing, the development methodology, and the appropriate definition of the EHR architecture. In addition, these studies present characteristics and the main technologies that can contribute to research on the development of EHR, taking into account the Brazilian scenario.

In this context, the implementation of EHR adhering to the most comprehensive interoperability standards will increase the capacity of health services regarding the basic principles of PHC, such as access, longitudinality, integrality, and care coordination. Nevertheless, Brazil needs to accelerate and support initiatives that are developing actions to promote the deployment of EHR in a coordinated manner, in the three spheres of executive power, as well as interoperability between public and private services.

## Data availability statement

The original contributions presented in the study are included in the article/supplementary material, further inquiries can be directed to the corresponding author/s.

## Author contributions

IB, FF, and DB: collection, organizing, and review of the literature. IB, FF, and RV: preparing the manuscript and editing and revision. DB, JP, JH, AM, KC, GC, and AC: manuscript review and modification. All authors contributed to the review of the paper and approved the submitted version.

## Funding

This study was supported by Brazil's Ministry of Health.

## Conflict of interest

The authors declare that the research was conducted in the absence of any commercial or financial relationships that could be construed as a potential conflict of interest.

## Publisher's note

All claims expressed in this article are solely those of the authors and do not necessarily represent those of their affiliated organizations, or those of the publisher, the editors and the reviewers. Any product that may be evaluated in this article, or claim that may be made by its manufacturer, is not guaranteed or endorsed by the publisher.
